# Preoperative Sleep Patterns and Their Impact on Outcomes in Total Hip and Knee Replacement: An Observational Study

**DOI:** 10.7759/cureus.82253

**Published:** 2025-04-14

**Authors:** Yousif Mohamed, Conor O'Driscoll, Madalena Nina Rente, Muhammad Bilal, May S Cleary, Fiachra Rowan

**Affiliations:** 1 Orthopedics, University Hospital Waterford, Waterford, IRL; 2 Orthopedics, Kilcreene Regional Orthopaedic Hospital, Kilkenny, IRL; 3 Orthopedics, University College Cork, Cork, IRL

**Keywords:** orthopedics surgery outcomes, pittsburgh sleep quality index (psqi), postoperative outcomes, preoperative sleep, total joint replacement

## Abstract

Background

Researching modifiable preoperative risk factors is essential for improving outcomes following total joint replacement (TJR). This study explores whether preoperative sleep performance influences pain and recovery in the early postoperative period.

Methods

This prospective observational study was conducted at an academic elective orthopedic hospital, recruiting patients undergoing total hip replacement (THR) and total knee replacement (TKR). Preoperative sleep was assessed using the Pittsburgh Sleep Quality Index (PSQI). Measured outcomes included pain, oral morphine use, day of crutch mobility, independent bed transfer, and hospital length of stay.

Results

No statistically significant associations were found between preoperative PSQI scores and primary outcomes, although sex differences existed in THR patients regarding early postoperative pain. The correlation between PSQI and hospital stay was weakly positive for THR (r = 0.223, p = 0.082) and negligible for TKR (r = 0.041, p = 0.807). PSQI showed no significant correlation with early mobility (THR: r = 0.111, p = 0.391; TKR: r = 0.115, p = 0.491) or postoperative morphine use (THR: r = 0.108, p = 0.403; TKR: r = 0.170, p = 0.309). Female THR patients had higher pain scores on days 0 and 1 and poorer PSQI scores.

Conclusions

Preoperative sleep hygiene was not associated with hospital stay, mobility, or pain in the immediate postoperative period after TJR. However, sleep may impact long-term recovery, highlighting the need for further research on modifiable preoperative factors and sex differences in post-TJR rehabilitation.

## Introduction

With the increasing demand for total knee replacement (TKR) and total hip replacement (THR) surgeries, the medical community is recognizing that the success of these procedures is affected by various factors, some of which go beyond the usual physiological and surgical aspects [1,2]. Joint arthroplasty surgeries have undergone significant advancements in recent years, providing remarkable relief for individuals struggling with debilitating joint conditions [3,4]. Despite these advancements, optimizing the factors contributing to successful outcomes remains an ongoing challenge [5]. Optimizing a variety of modifiable factors [6], including preoperative lifestyle and health-related habits, has become essential in improving recovery outcomes and enhancing patient satisfaction and has gained traction in popular culture with electronic methods to measure and record many factors available. Factors such as nutrition [7], physical activity [8], mental well-being [9], and sleep quality are now integral parts of preoperative care. One such factor that has garnered increasing interest is the impact of preoperative sleep hygiene on surgical outcomes [10,11].

Studies have shown that poor sleep quality before surgery can negatively affect the body’s healing processes, immune function [12], and pain perception [13]. Research indicates that patients with poor preoperative sleep tend to experience higher levels of postoperative pain, slower recovery, and increased risk of complications, as sleep disruptions are known to increase inflammatory responses [14]. Additionally, inadequate sleep may compromise mental resilience, contributing to anxiety, depression, and decreased patient satisfaction with recovery [15]. In orthopedic surgery, where functional recovery and pain management are critical to achieving successful outcomes, sleep hygiene is emerging as a significant modifiable factor [10]. A thorough analysis of all possible factors is necessary to fully comprehend patient care. According to one study, maintaining good sleep habits before surgery may significantly impact the patient's recovery and level of satisfaction after the procedure [16].

We hypothesized that poorer preoperative sleep performance, as measured by a validated tool, has a negative influence on early postoperative outcomes. We aimed to correlate sleep with pain, opiate requirement, mobility, and length of stay (LOS).

## Materials and methods

Patient enrollment and eligibility criteria

A prospective observational study involving patients undergoing total joint replacement (TJR) was conducted at an academic orthopedic unit. The aim of the study was to determine whether preoperative sleep hygiene influences immediate postoperative outcomes following TJR.

Sleep assessment

Preoperative sleep patterns were assessed using the Pittsburgh Sleep Quality Index (PSQI). This validated instrument measures sleep quality over four weeks prior to admission. Nineteen individual items generate seven “component” scores: subjective sleep quality, sleep latency, sleep duration, habitual sleep efficiency, sleep disturbances, use of sleeping medication, and daytime dysfunction. The sum of the scores for these seven components yields one global score, with a maximum score of 21 [17]. A higher PSQI score indicates poorer sleep quality. A difference of three points is considered clinically significant in sleep performance. Based on this, a sample size of 100 consecutive patients was determined to provide adequate power for the study.

Postoperative outcome measures

Pain was recorded using the Visual Analogue Scale (VAS) on the admission day and at subsequent postoperative intervals. The VAS is a validated tool consisting of a 10 cm line representing a continuum from “no pain” to “worst pain imaginable” [18]. Morphine requirements were calculated according to preparations administered; for example, 10 mg of OxyContin (Purdue Pharma L.P., Stamford, CT, United States) equaled 15 mg of morphine, and 5 mg of OxyNorm equaled 7.5 mg of morphine. At our institution, patients’ pain control follows the Enhanced Recovery After Surgery guidelines [19]. A multimodal approach was adopted, incorporating oral analgesic medications such as paracetamol, NSAIDs, and slow-release opioids regularly once patients resumed postoperative oral intake. Additionally, fast-release opioids were administered as needed, typically at intervals of four to six hours. This structured pain management strategy aimed to address pain effectively while aligning with recognized standards of care. Day of independence in and out of bed postoperatively, time to mobility on crutches, and LOS were also recorded.

Demographic data recorded included sex, age, and BMI. Concomitant diseases were recorded, including hypertension, heart diseases (such as coronary artery disease and arrhythmia), diabetes, chronic bronchitis, migraine, and osteoporosis.

Inclusion criteria

We included patients who were undergoing unilateral elective primary TJR, older than 18 years, and demonstrated the willingness and ability to complete questionnaire surveys. Those patients undergoing revision surgery, bilateral TJR, or unicompartmental knee replacement or demonstrating regular use of sleep-related medication who had neurological diseases or psychiatric disorders and were unwilling to participate in the study, had communication barriers, and had a LOS of more than five days for reasons related to logistical or social factors related to discharge destination were excluded.

Surgical procedure

We performed THR and TKR surgeries under spinal anesthesia with appropriate thromboprophylaxis and chemoprophylaxis. The surgical approach to the hip and the type of implant used for THR and TKR varied. Direct anterior, direct lateral, and posterior approaches were used according to surgeon preference. The surgeon performed hybrid or cementless THRs and mechanically aligned or robotically assisted kinematically aligned TKRs according to his or her preference.

Data collection

Before administering preoperative medication upon admission, we instructed patients to complete the PSQI [17]. Concurrently, the VAS score was recorded on the admission day and at subsequent postoperative intervals - one day, two days, three days, and four days - from the patient’s observational records. We recorded the LOS per 24-hour period and mobility on crutches from the patients' charts as determined by the physiotherapist.

Statistical analysis

We examined the distribution of demographic data, baseline data, and primary and secondary outcomes. For quantitative variables, we used measures of central tendency (mean, SD), and for qualitative variables, we used percentages. Pearson correlations were performed between the PSQI score and the VAS score, morphine requirement, day of crutch mobility, time to get in and out of bed independently, and length of hospital stay. We used independent sample t-tests to compare the means of the comparisons. We conducted all the data analyses using IBM SPSS Statistics for Windows, Version 20.0 (Released 2011; IBM Corp., Armonk, NY, USA). The significance level was set at p < 0.05.

Ethical statement

The Institutional Review Board at University Hospital Waterford/Kilcreene Group looked over the study and confirmed that it did not need formal ethical approval because it did not include any treatments, identifiable patient information, or procedures that needed official oversight. This review ensured adherence to ethical standards and protection of patient confidentiality.

## Results

Demographic and surgical characteristics of the study cohort

The study cohort (N = 100) included a diverse range of patients undergoing THR and TKR. Most patients were over 60 years old (64 (64.0%)), followed by those aged 40-60 (33 (33.0%)) and a smaller group under 40 years (3 (3.0%)). The gender distribution was nearly balanced, with 51 (51.0%) male and 49 (49.0%) female. In terms of BMI, 47 (47.0%) were classified as obese, 40 (40.0%) as overweight, and 13 (13.0%) had normal weight (Table 1).

**Table 1:** Demographic characteristics of patients undergoing TKR and THR THR: total hip replacement; TKR: total knee replacement

Parameter	Category	Frequency	Percentage
Age	<40 years	3	3.0
	40-60 years	33	33.0
	>60 years	64	64.0
Gender	Male	51	51.0
	Female	49	49.0
BMI	Normal weight	13	13.0
	Overweight	40	40.0
	Obese	47	47.0
Type of surgery	THR	62	62.0
	TKR	38	38.0

Analysis of preoperative sleep hygiene by demographic and surgical subgroups

Preoperative sleep quality, measured by the PSQI, was assessed across different demographic and surgical subgroups. The mean PSQI score for patients undergoing THR was 8.6 ± 3.8, while for TKR patients, it was 8.4 ± 3.3, with no significant difference between the two (p = 0.787). Females had higher PSQI scores (9.2 ± 3.6) compared to males (7.8 ± 3.5), indicating poorer preoperative sleep hygiene among female patients (p = 0.048). There were no significant differences in PSQI scores across age groups or BMI categories (Table 2).

**Table 2:** Mean preoperative PSQI scores by demographic and surgical subgroups PSQI: Pittsburgh Sleep Quality Index; THR: total hip replacement; TKR: total knee replacement * Statistically significant (p < 0.05)

Variable	Subgroup	PSQI (Mean ± SD)	p-Value
Type of joint replacement	THR	8.6 ± 3.8	0.787
	TKR	8.4 ± 3.3	
Gender	Male	7.8 ± 3.5	0.048*
	Female	9.2 ± 3.6	
Age (years)	<40	9.0 ± 5.2	0.541
	40-60	9.1 ± 3.4	
	>60	8.2 ± 3.6	
BMI	Normal weight	8.0 ± 3.7	0.582
	Overweight	8.7 ± 3.8	
	Obese	8.6 ± 3.4	

Correlation between preoperative sleep hygiene and length of hospital stay in THR and TKR patients

The relationship between preoperative PSQI scores and LOS was analyzed for both THR and TKR patients. For the THR group, there was a weak positive correlation between PSQI scores and LOS (r = 0.223), which approached statistical significance (p = 0.082). This suggests that poorer preoperative sleep hygiene may be associated with a slightly longer hospital stay, although the correlation was not statistically significant. In contrast, for the TKR group, the correlation was negligible (r = 0.041) and nonsignificant (p = 0.807), indicating no meaningful relationship between PSQI scores and LOS in these patients (Table 3).

**Table 3:** Correlation between LOS and PSQI scores in THR and TKR patients LOS: length of stay; PSQI: Pittsburgh Sleep Quality Index; THR: total hip replacement; TKR: total knee replacement

Outcome	THR		TKR	
	r-Value	p-Value	r-Value	p-Value
LOS	0.223	0.082	0.041	0.807

Impact of preoperative sleep hygiene on postoperative opioid requirements in THR and TKR patients

The correlation between preoperative PSQI scores and postoperative opioid analgesia requirements, measured by conversion to oral morphine, was assessed for both THR and TKR patients. In the THR group, the correlation was weak (r = 0.108) and not statistically significant (p = 0.403), indicating that preoperative sleep hygiene did not significantly influence the need for postoperative oral morphine. Similarly, in the TKR group, a weak correlation was observed (r = 0.170), which was also nonsignificant (p = 0.309). These findings suggest that preoperative PSQI scores had a limited impact on the requirement for postoperative opioid analgesia in both groups (Table 4).

**Table 4:** Correlation between PSQI scores and opioid analgesia requirements in THR and TKR patients PSQI: Pittsburgh Sleep Quality Index; THR: total hip replacement; TKR: total knee replacement

Outcome	THR		TKR	
	r-Value	p-Value	r-Value	p-Value
Opioid analgesia requirements	0.108	0.403	0.17	0.309

Association between preoperative sleep quality and early postoperative mobility in THR and TKR patients

The relationship between preoperative PSQI scores and early postoperative mobility on crutches was analyzed for both THR and TKR patients. In the THR group, the correlation was weak (r = 0.111) and not statistically significant (p = 0.391), indicating that preoperative sleep quality did not have a significant impact on early crutch mobility. Similarly, for the TKR group, a weak and nonsignificant correlation was observed (r = 0.115, p = 0.491). These results suggest that preoperative PSQI scores were not associated with differences in early postoperative mobility on crutches for patients in either group (Table 5).

**Table 5:** Correlation between mobility on crutches and PSQI scores in THR and TKA patients PSQI: Pittsburgh Sleep Quality Index; THR: total hip replacement; TKR: total knee replacement

Outcome	THR		TKR	
	r-Value	p-Value	r-Value	p-Value
Mobility on crutches	0.111	0.391	0.115	0.491

## Discussion

This study aimed to investigate the relationship between preoperative sleep hygiene and postoperative outcomes in patients undergoing either THR or TKR. Our findings provide insight into the impact of preoperative sleep quality on various postoperative parameters, including pain, LOS, day of independence, mobility on crutches, and morphine usage. These results were compared with previous studies in order to place our findings within the existing body of knowledge.

Our study demonstrated that preoperative sleep quality, as measured by the PSQI, did not significantly correlate with length of hospital stay, early mobility on crutches, day of independence in and out of bed, or the requirement for oral morphine in either THR or TKR patients, but sex differences existed. These findings align with some aspects of the study by Luo et al., which revealed significant correlations between preoperative sleep quality and postoperative outcomes, including pain and functional scores in TJA patients [1]. However, our results diverge in that we did not observe a significant impact of preoperative sleep on LOS or early mobility, suggesting that other factors might mitigate the influence of sleep quality in our patient cohort.

A study by Bjurström et al. highlighted that poor preoperative sleep quality predicted higher long-term postoperative pain severity and increased opioid usage immediately after surgery in THR patients [2]. In contrast, our findings did not show a significant correlation between PSQI scores and postoperative pain levels at any time point for either THR or TKR patients. This discrepancy could be attributed to differences in study design, sample size, and patient populations. Bjurström et al. included a more focused cohort of patients with disabling osteoarthritis pain who underwent THR, which might have accentuated the impact of preoperative sleep disturbances on postoperative pain outcomes. Additionally, our study period was shorter than that of the referenced study, which might have influenced the differences in findings.

Furthermore, the gender differences observed in our study, where females reported significantly higher PSQI scores and pain levels postoperatively, particularly in the early days following surgery, are consistent with broader literature [20] and indicate that women often experience greater pain sensitivity and poorer sleep quality compared to males. These sex-specific findings underscore the need for tailored preoperative interventions to address sleep disturbances, particularly in female patients, to improve postoperative pain management and overall recovery.

Our study’s results, such as the impact of the PSQI on LOS and early mobility, suggest that preoperative sleep quality alone may not be a robust predictor of these outcomes. These findings align with Luo et al.’s assertion that while preoperative sleep quality is correlated with certain postoperative outcomes, it is one of many factors influencing recovery. Comprehensive preoperative assessments considering multiple variables, including psychological, social, and physical health factors, are essential for predicting and enhancing postoperative outcomes [21,22].

Limitations

This study has several limitations. First, the surgical approach to the hip may influence immediate postoperative recovery; however, we did not stratify patients by approach due to the potential dilutional effect on the dataset. We acknowledge this as a possible confounding factor [23,24]. We did not stratify our data according to surgical approach due to the dilutional effect this may have on the data set but recognize this as a possible confounding factor in the study. Second, robotic-assisted surgery and varying alignment philosophies in TKR may have influenced early postoperative outcomes but were not controlled for in this analysis [25]. Third, although psychological factors such as pain catastrophizing, anxiety, and resilience are known to affect pain perception and recovery [26], we did not include formal assessments of these variables in our cohort. Future studies incorporating these elements could provide a more comprehensive understanding of the role of preoperative sleep in postoperative outcomes.

## Conclusions

While preoperative sleep quality assessed by the PSQI was not significantly correlated with several key postoperative outcomes in our study, the observed trends and sex differences warrant further investigation. Future studies with larger sample sizes, stratified analyses, and interventions aimed at improving sleep hygiene preoperatively are recommended to elucidate the complex relationship between sleep and surgical recovery. Our findings contribute to the ongoing dialogue on optimizing preoperative care to enhance surgical outcomes and patient satisfaction in orthopedic surgery.
